# Two cases of intraoperative hemodynamic instability during combined thoracoscopic-laparoscopic surgery for esophagogastric junction carcinoma

**DOI:** 10.1186/s40981-021-00419-x

**Published:** 2021-02-10

**Authors:** Makiko Tani, Yoshikazu Matsuoka, Mayu Sugihara, Ayaka Fujii, Tomoyuki Kanazawa, Hiroshi Morimatsu

**Affiliations:** 1 Department of Anesthesiology and Resuscitology, Graduate School of Medicine Dentistry and Pharmaceutical Sciences, Okayama University, 2-5-1, Shikata-cho, Kita-ku, Okayama, 700-8558 Japan; 2 Department of Anesthesiology and Resuscitology, Okayama University Hospital, 2-5-1, Shikata-cho, Kita-ku, Okayama, 700-8558 Japan

**Keywords:** Esophagogastric junction carcinoma, Intra-mediastinal valvuloplastic esophagogastrostomy, Hemodynamic instability, Tension pneumothorax, Anesthetic management

## Abstract

**Background:**

Intraoperative complications during combined thoracoscopic-laparoscopic surgery for esophagogastric junction (EGJ) carcinoma have not been reported as compared to those during surgery for esophageal carcinoma. We present two cases which had surgery-related hemodynamic instability during laparoscopic proximal gastrectomy and intra-mediastinal valvuloplastic esophagogastrostomy (vEG) with thoracoscopic mediastinal lymphadenectomy for EGJ carcinoma.

**Case presentation:**

In case 1, the patient fell into hypotension with hypoxemia during laparoscopic vEG due to pneumothorax caused by entry of intraabdominal carbon dioxide. In case 2, ventricular arrythmia and ST elevation occurred during laparoscopic vEG. Pericardium retraction to secure surgical field during reconstruction compressed the coronary artery, which caused coronary malperfusion. These two events were induced by the surgical procedure, characterized by the following: (1) connection of the thoracic and abdominal cavities and (2) cardiac displacement during vEG.

**Conclusion:**

These cases indicated tension pneumothorax and coronary ischemia are possible intraoperative complications specific to combined thoracoscopic-laparoscopic surgery for EGJ carcinoma.

## Background

Intraoperative cardiac complications during surgery for esophageal cancer, regardless of being performed by thoracotomy or by thoracoscopy, are not rare and have been previously reported [1–4]. However, no reports have been published describing specific intraoperative cardiac complications during surgery for esophagogastric junction (EGJ) carcinoma as far as we have known.

In our institution, standard procedure for EGJ carcinoma is lower esophagectomy and proximal gastrectomy with lower mediastinal lymphadenectomy followed by valvuloplastic esophagogastrostomy (vEG) [5], which is performed by combined thoracoscopic laparoscopic approach. In this surgical technique, mediastinal lymphadenectomy and lower esophagus mobilization were performed by right thoracoscopic approach under one lung ventilation in prone position. Subsequently, abdominal lymphadenectomy, esophago-proximal gastrectomy, and reconstruction are laparoscopically performed in a supine position. This procedure has the following features: (1) bilateral thoracic cavities and abdominal cavity are connected and (2) cardiac displacement is necessary during valvuloplasty.

In this case report, we describe two cases in which hemodynamic instability occurred associated with the procedure and discuss anesthetic management.

## Case presentation

### Case 1

A 57-year-old male with chronic obstructive lung disease was diagnosed with EGJ adenocarcinoma. There was no bulla in his preoperative chest computed tomography (CT). Laparoscopic proximal gastrectomy combined with transhiatal lower esophagectomy and thoracoscopic mediastinal lymphadenectomy followed by intra-mediastinal vEG were scheduled. General anesthesia with propofol, remifentanil, and rocuronium was provided with thoracic epidural anesthesia (T7/8). The trachea was intubated with a single lumen tube, and a bronchial blocker was inserted in the right main bronchus for one-lung ventilation.

Following anesthesia induction, the patient was turned to the prone position. Intrathoracic procedure was performed thoracoscopically via the right thorax under one-lung ventilation. After mediastinal lymphadenectomy and esophageal mobilization, the bilateral pleural spaces were connected. A 19 French thoracic tube was placed in the right pleural cavity, and both-lung ventilation was restarted at the end of the intrathoracic procedure.

The patient was then turned to the supine position. The laparoscopic procedure was started with lymphadenectomy following proximal gastrectomy. In this phase, the left crus of diaphragm was incised and esophageal hiatus was opened. The abdominal cavity and bilateral thorax were then connected. After cutting off the lower esophagus, an esophago-proximal gastrectomy was performed.

After 15 min resumption of carbon dioxide (CO_2_) inflation for reconstruction, peripheral arterial oxygen saturation (SpO_2_) gradually dropped from 99 to 94% under fraction of inspired oxygen (F_I_O_2_) 0.4, and tachycardia (heart rate 110 beats per minute) and hypotension (75/50 mmHg) appeared. Central venous pressure raised from 8 to 14 mmHg. Tidal volume simultaneously decreased from 400 to 200 ml under pressure control ventilation. We found that massive air leakage was continuously drained from the right thoracic tube independently of respiratory cycle.

We deduced that this hypoventilation and hemodynamic instability were attributed to tension pneumothorax by CO_2_ inflation. CO_2_ entered the bilateral pleural cavity via the peritoneum—left pleural cavity—right pleural cavity connection. However, the inflation management system continued providing a large amount of CO_2_ to keep pneumoperitoneum pressure (10 cmH_2_O). To treat the tension pneumothorax of this patient, stopping pneumoperitoneum and converting to laparotomy was a possible option; however, we did not stop pneumoperitoneum until the esophageal-remnant stomach anastomosis was accomplished. The reason was that the anastomosis required advanced skills if performed even by laparoscopy, much more by laparotomy. The lower esophagus was transected in the mediastinum, and the manipulation had to be performed deep in the mediastinum. In addition, thoracoabdominal incision was necessary if we stopped laparoscopic surgery. Therefore, surgeons requested to continue laparoscopic anastomosis as far as possible. In order to improve patient’s oxygenation, we raised positive end-expiratory pressure (PEEP) from 5 to 12 cmH_2_O and performed a lung recruitment maneuver to reopen the collapsed lung, and we increased tidal volume to 500 ml to maintain adequate ventilation. To maintain hemodynamic stability, we administered norepinephrine until the reconstruction was finished. The patient’s oxygenation, SpO_2_ and blood pressure returned to 100% and 110 mmHg, respectively. At the conclusion of the surgery, the second thoracic drain was inserted into the left pleural cavity. Confirming that the patient was hemodynamically stable and that no pneumothorax or atelectasis remained in the chest X-ray, the patient was extubated and transferred to the intensive care unit (ICU). The patient spent another uneventful day in the ICU and was discharged at postoperative day 17. The chest CT on post-operative day 3 showed no bulla or pneumothorax.

### Case 2

A 63-year-old male with no other past history except smoking was diagnosed with EGJ adenocarcinoma. The same surgery as case 1 was scheduled. Basically, anesthesia and airway management strategies were the same as that of case 1.

**Fig. 1:**
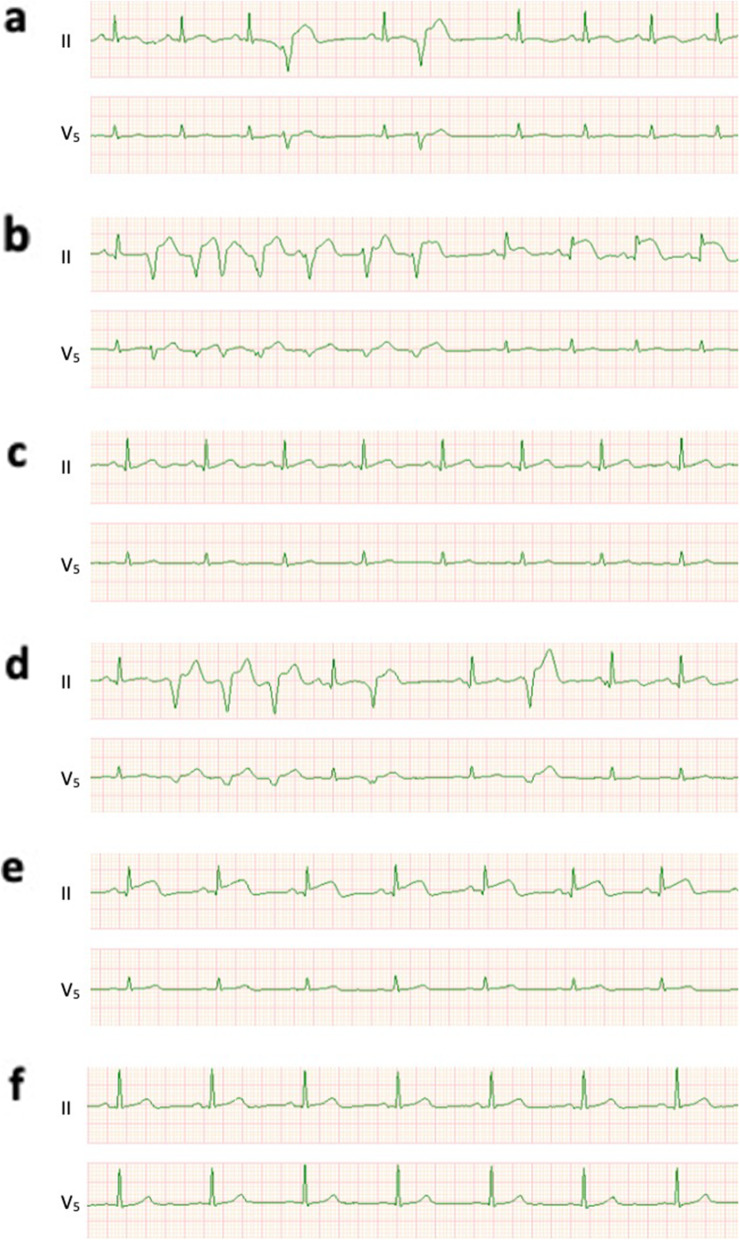
Electrocardiogram waveform changes in case 2. **a** Paroxysmal ventricular contractions (PVCs) emerged after starting the esophagogastrostomy. **b** Non-sustained ventricular tachycardia (VT) and ST elevation in lead II. **c** VT and ST elevation temporally disappeared after adjusting the position of the pericardium retractor and administering nicorandil. **d** PVCs reoccurred with ST elevation in lead II. **e** Persistent ST elevation in lead II after the pericardium retraction release. **f** Normal electrocardiogram in the end of surgery

The patient was stable during the thoracic and abdominal phases before laparoscopic intra-mediastinal vEG. After starting the esophagogastrostomy, paroxysmal ventricular contractions (PVCs) emerged (Fig. 1a). The PVCs then converted to non-sustained ventricular tachycardia with ST elevation in electrocardiogram (ECG) lead II (Fig. 1b). We conjectured the ECG change occurred due to impaired coronary perfusion by pericardium retraction when securing the surgical field. By adjusting the position of the pericardium retractor and administering nicorandil (4 mg/h) to maintain coronary perfusion, ECG temporarily returned to normal levels (Fig. 1c). Once the pericardium retraction became aggressive, frequent PVCs recurred with ST elevation in lead II (Fig. 1d). The ST elevation persisted after the pericardium retraction was released (Fig. 1e). Systolic blood pressure dropped to 80 mmHg. We started noradrenaline infusion (0.04 mcg/kg/h), thereby raising blood pressure and coronary perfusion pressure. ST elevation gradually improved and returned to normal levels in one hour (Fig. 1f). The surgery was performed as scheduled. The patient was transferred to the ICU under sedation and tracheal intubation. In the ICU, a 12-lead ECG showed no abnormalities including ST elevation or arrhythmia. Creatine kinase (CK) was 394 units/L, indicating a slight elevation (normal range: 59–248 units/L) with no elevation of CK-myocardial band. Troponin T was 0.286 ng/mL (normal range: 0–0.014 ng/mL). There was a possibility of transient microischemia due to transient epicardial coronary occlusion or vasospasm; however, there was no evidence of persistent cardiac infarction. We did not perform a coronary angiography. The patient was extubated the day after surgery and discharged after 11 days.

## Discussion

Minimally invasive surgeries for esophageal cancer or EGJ carcinoma are less invasive than open surgeries, and a systematic review reported that minimally invasive procedures have lower pulmonary complications compared with open procedures [6]. Valvuloplastic esophagogastrostomy with double flap technique prevents gastroesophageal reflux and anastomotic stenosis [5, 7]. However, to complete this anastomosis laparoscopically, cutting the diaphragm and opening the left mediastinal pleura are necessary for making a large space [5]. These additional surgical incisions enlarged both the thorax and abdominal cavities’ connection, which was already made during thoracic phase and proximal gastrectomy. In addition, a complicated anastomosis in a small space was required. These surgical properties were related to adverse events in the two cases.

The major cause of hemodynamic instability in case 1 was tension pneumothorax. Bilateral pleural cavity connection in the thoracic phase and the left diaphragm incision in the abdominal phase caused CO_2_ inflow to the bilateral pleural cavity during pneumoperitoneum. In this case, we could diagnose that the desaturation and hemodynamic instability were due to a tension pneumothorax when massive gas leakage occurred. The gas leakage was not relevant to the respiratory cycle. These facts indicated that the pneumothorax was not due to lung injury under positive pressure ventilation. In fact, there was no bulla or pneumothorax pre- and post-operative chest CT. Unlike this case, there is a possibility that the CO_2_ could not be drained by a thoracic tube in case of tube malposition. In such a case, cardiorespiratory deterioration could progress more drastically.

To accomplish this surgery, pneumoperitoneum for intra-mediastinal esophagogastrostomy is necessary, and pneumothorax is inevitable. Anesthesiologists have no choice but to accept some cardiorespiratory instability. Hemodynamic instability in pneumothorax is caused by decreased venous return due to high intrathoracic pressure. In this case, high PEEP to release the lung from atelectasis might aggravate the hypotension by raising intrathoracic pressure. To balance obtaining a surgical view while keeping hemodynamic stability, a sufficient amount of CO_2_ supply for pneumoperitoneum with thoracic drainage and adequate intravenous fluids are necessary. Regarding respiratory management, anesthesiologists should accept high peak inspiratory pressure to obtain tidal volume for adequate ventilation and oxygenation. We usually accept peak inspiratory pressure below 30 cmH_2_O during artificial CO_2_ pneumothorax (10 cmH_2_O) in order to keep transpulmonary pressure below 20 cmH_2_O and to prevent lung injury [8]. We cannot measure esophageal pressure in this surgery, and we have no choice but to estimate transpulmonary pressure. Of course, lower inspiratory pressure should be better for patients with the vulnerable lungs. Therefore, surgical tolerance for this procedure should be prudently considered in patients with a high risk of developing pneumothorax (e.g., having large bulla and severe emphysema) and in patients with impaired cardiac function.

**Fig. 2:**
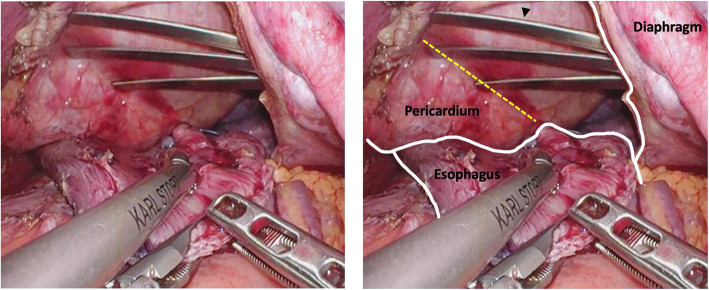
Intraoperative laparoscopic findings at the beginning of esophagogastric anastomosis. The left diaphragm is incised, and the pericardium is lifted up by the retractor (indicated by the arrowhead) to expose the surgical field. The retractor blade is placed close to the posterior interventricular sulcus (indicated by yellow dotted line)

In case 2, ventricular tachycardia and ST elevation in ECG appeared after starting the esophagogastrostomy. For laparoscopic intra-mediastinal vEG, pericardial retraction is necessary to ensure the surgical filed. The right coronary artery descends in the right atrioventricular groove and then runs downward in the posterior interventricular sulcus to the apex (Fig. 2). The right coronary artery—which runs directly above the left diaphragm incision—was compressed by the pericardial retractor, and the right coronary flow suppression induced ST elevation in lead II and arrhythmia. Administration of nicorandil and norepinephrine and compression release improved coronary blood flow. We could prevent the ischemic change from becoming permanent.

Here, we presented tension pneumothorax and cardiac ischemia as intraoperative complications associated with laparoscopic proximal gastrectomy and intra-mediastinal vEG with thoracoscopic mediastinal lymphadenectomy for EGJ carcinoma. Regarding postoperative complications associated with this procedure, Umeki et al. reported a case of cardiac tamponade on postoperative day 1 [9]. The cause was intraoperative needle injury to the pericardium during vEG. In the report, the authors discussed that heat injury to the pericardium by an energy device can cause perioperative cardiac tamponade.

## Conclusion

Tension pneumothorax and cardiac ischemia are possible complications of combined thoracoscopic-laparoscopic surgery for EGJ carcinoma. More case accumulations are necessary to reveal complications associated with the surgical procedure and to safely perform the surgery.

## Data Availability

Not applicable due to patient privacy concerns.

## Abbreviations

EGJ: Esophagogastric junction
EG: Esophagogastrostomy
vEG: Valvuloplastic esophagogastrostomy
CT: Computed tomography
CO_2_: Carbon dioxide
SpO_2_: Peripheral arterial oxygen saturation
F_I_O_2_: Fraction of inspired oxygen
PEEP: Positive end-expiratory pressure
ICU: Intensive care unit
PVC: Paroxysmal ventricular contraction
ECG: Electrocardiogram
CK: Creatine kinase
